# CBP Is Required for Establishing Adaptive Gene Programs in the Adult Mouse Brain

**DOI:** 10.1523/JNEUROSCI.0970-22.2022

**Published:** 2022-10-19

**Authors:** Michal Lipinski, Sergio Niñerola, Miguel Fuentes-Ramos, Luis M. Valor, Beatriz del Blanco, Jose P. López-Atalaya, Angel Barco

**Affiliations:** Instituto de Neurociencias, Consejo Superior de Investigaciones Científicas, Universidad Miguel Hernández, Campus de Sant Joan, 03550 Alicante, Spain

**Keywords:** activity-driven transcription, CBP, intellectual disability, lysine acetylation, neuroepigenetics, p300

## Abstract

Environmental factors and life experiences impinge on brain circuits triggering adaptive changes. Epigenetic regulators contribute to this neuroadaptation by enhancing or suppressing specific gene programs. The paralogous transcriptional coactivators and lysine acetyltransferases CREB binding protein (CBP) and p300 are involved in brain plasticity and stimulus-dependent transcription, but their specific roles in neuroadaptation are not fully understood. Here we investigated the impact of eliminating either CBP or p300 in excitatory neurons of the adult forebrain of mice from both sexes using inducible and cell type-restricted knock-out strains. The elimination of CBP, but not p300, reduced the expression and chromatin acetylation of plasticity genes, dampened activity-driven transcription, and caused memory deficits. The defects became more prominent in elderly mice and in paradigms that involved enduring changes in transcription, such as kindling and environmental enrichment, in which CBP loss interfered with the establishment of activity-induced transcriptional and epigenetic changes in response to stimulus or experience. These findings further strengthen the link between CBP deficiency in excitatory neurons and etiopathology in the nervous system.

**SIGNIFICANCE STATEMENT** How environmental conditions and life experiences impinge on mature brain circuits to elicit adaptive responses that favor the survival of the organism remains an outstanding question in neurosciences. Epigenetic regulators are thought to contribute to neuroadaptation by initiating or enhancing adaptive gene programs. In this article, we examined the role of CREB binding protein (CBP) and p300, two paralogous transcriptional coactivators and histone acetyltransferases involved in cognitive processes and intellectual disability, in neuroadaptation in adult hippocampal circuits. Our experiments demonstrate that CBP, but not its paralog p300, plays a highly specific role in the epigenetic regulation of neuronal plasticity gene programs in response to stimulus and provide unprecedented insight into the molecular mechanisms underlying neuroadaptation.

## Introduction

Activity-driven transcriptional and epigenetic mechanisms serve many processes in the adult brain, including the modulation of synaptic plasticity and memory, and the adaptation to environmental conditions (Gräff and Tsai, 2013; Lopez-Atalaya and Barco, 2014; Yap and Greenberg, 2018; Fuentes-Ramos et al., 2021). These mechanisms can provide a molecular substrate for enduring changes in the behavior of an animal by fine-tuning gene expression according to the neuronal activation history (Fernandez-Albert et al., 2019; Marco et al., 2020). The ubiquitous detection of transcriptional dysregulation and chromatin alterations in disorders associated with cognitive impairments and maladaptive behaviors further highlights the relevance of these mechanisms in cognition (Bjornsson, 2015; Nestler et al., 2016).

Studies in humans and rodents have strongly linked the activity of the transcriptional coactivators CREB binding protein (CBP) and p300 with cognitive processes (Lopez-Atalaya et al., 2014). Both paralogous proteins have intrinsic lysine acetyltransferase (KAT) activity and are also known as KAT3A and KAT3B, respectively. Whereas the germinal loss of either of these proteins leads to early embryonic death (Yao et al., 1998), hemizygous mutations in the genes encoding for CBP and p300 are linked in humans to a rare intellectual disability disorder known as Rubinstein–Taybi syndrome (RSTS; Petrij et al., 1995; Roelfsema et al., 2005), and cause cognitive impairments and other syndromic features in mice (Alarcón et al., 2004; Viosca et al., 2010). Pharmacological experiments and genetic studies in which CBP is specifically eliminated during development or in the early postnatal brain have later shown that cognitive impairments are not exclusively derived from developmental defects and pointed to a role for these proteins in neuronal plasticity in the adult brain (Chen et al., 2010; Barrett et al., 2011; Valor et al., 2011). However, the gene ablation strategies used in those studies produced mosaic recombination or triggered gene ablation in young neurons that are part of immature circuits still undergoing pruning and refinement (Semple et al., 2013), two processes in which CBP plays a critical role (del Blanco et al., 2019). These confounding factors complicate the interpretation of adult-specific functions. Compared with CBP, the impact of selectively eliminating p300 in forebrain neurons has been little investigated (Oliveira et al., 2011).

We have recently shown, using an efficient, inducible gene ablation approach, that CBP and p300 are jointly responsible for maintaining the active status of neuronal identity genes throughout life (Lipinski et al., 2020). Here, we used the same inducible gene ablation approach to investigate whether the individual elimination of CBP or p300 in forebrain neurons specifically compromised plasticity and adaptive processes in the adult brain. The combination of RNA-sequencing (seq) and ChIP-seq screens in the hippocampi of mice lacking CBP in excitatory neurons revealed deficits that reduced the transcriptional competence of genes linked to learning and memory. These transcriptional and epigenetic alterations correlated with impaired performance in several memory tasks. Furthermore, CBP-dependent gene dysregulation and behavioral deficits became more prominent in aged mice and in paradigms that involve a chronic or recurrent change in transcription induced by experience or environmental change. Comparative analysis with conditional knock-out mice for the CBP paralog p300 indicated that these functions are specific to CBP.

## Materials and Methods

### Mouse strains and treatments

**Figure 1.**
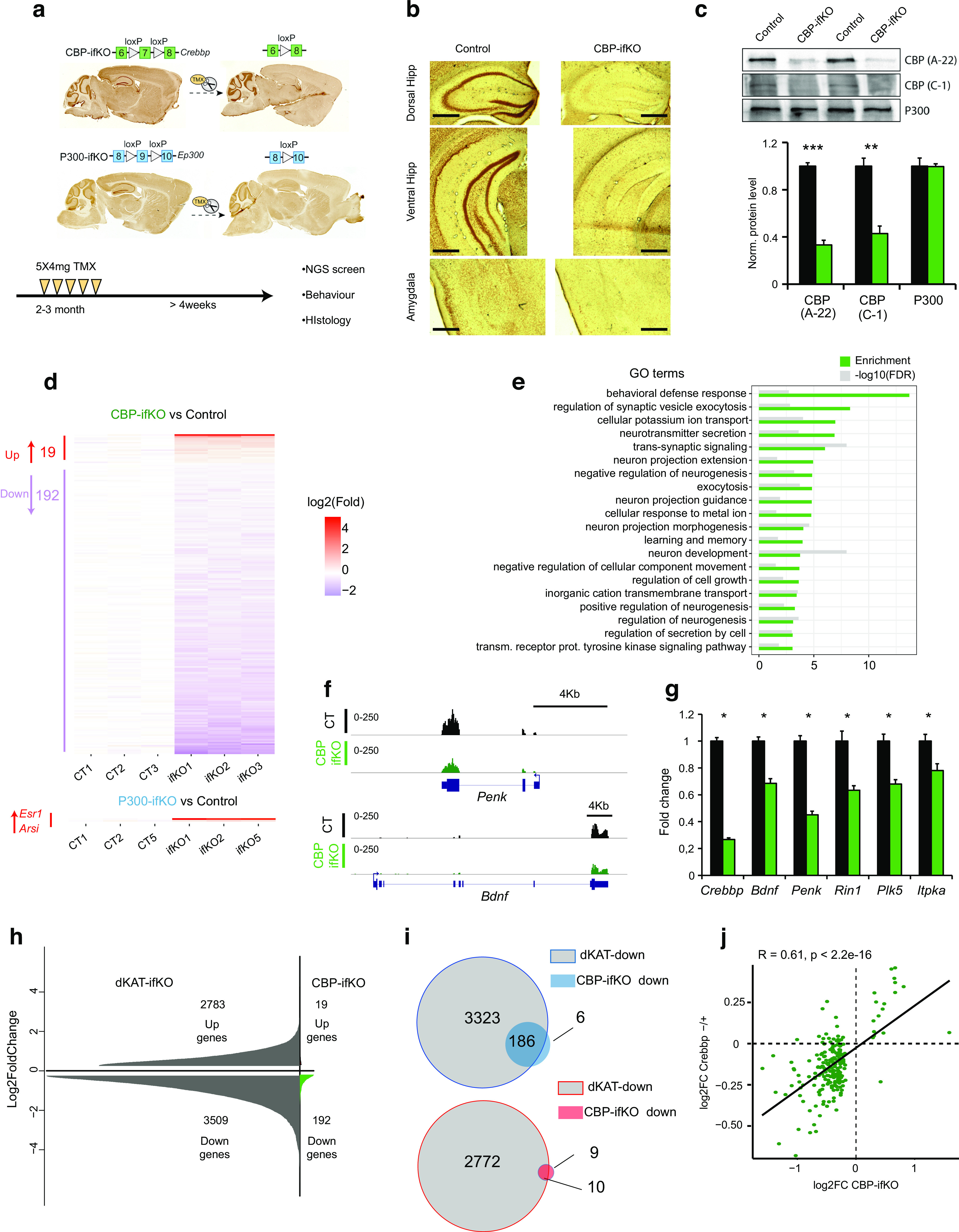
CBP, but not p300, is necessary for the normal expression of neuronal plasticity-related genes. ***a***, Scheme presenting the genetic strategy for the generation of CBP-ifKOs and p300-ifKOs illustrated with the immunostaining of sagittal brain slices of control and ifKOs that demonstrate the loss of CBP or p300 in areas where the Camk2a-creERT2 transgene is expressed. ***b***, IHC analysis demonstrated the loss of CBP in pyramidal and granular cells in the hippocampus of young adult (3- to 6-month-old) ifKO mice. Gene ablation is also appreciable in amygdala and cortex, but not in brain areas where the Cre recombinase is not expressed. Scale bar, 500 µm. ***c***, WB of CBP-ifKO (green bars) and control (WT, black bars) hippocampal protein extracts confirmed the loss of CBP expression in young adult (3- to 6-month-old) CBP-ifKOs using two different antibodies against CBP. No upregulation of p300 was observed. ***d***, RNA-seq was used for the differential expression profiling of CBP-ifKOs and control littermates (Extended Data Figs. 1-1, 1-2, additional details). Heatmap representations of upregulated and downregulated genes in young adult (3- to 6-month-old) CBP-ifKOs (top panel) and p300-ifKOs (bottom panel) referred to their respective control littermates. The creERT2 driver-related genes *Esr1* and *Arsi* are the only genes differentially expressed in p300-ifKOs. ***e***, GO analysis of downregulated genes in CBP-ifKOs. The top 20 GO biological process terms are shown. ***f***, IGV profiles of two representative genes downregulated in CBP-ifKOs. ***g***, qRT-PCR assays confirmed the downregulation of CBP target genes in the hippocampi of CBP-ifKOs. ***h***, Comparison of DEG sets in dKAT3-ifKOs (*p*-adjusted < 0.05; no fold cutoff) and CBP-ifKOs. ***i***, Overlap between the sets of genes downregulated in dKAT3-ifKOs (*p*-adjusted < 0.05; no cutoff) and CBP-ifKOs. ***j***, The comparison of differential expression profiles in CBP-ifKO and *Crebbp*^+/−^ mice revealed a gene dose effect and showed that many DEGs in CBP-ifKOs were also reduced in heterozygous mice, although did not reach the threshold for significance. *p-value < 0.05; **p-value < 0.01; ***p-value < 0.001.

The generation of *Crebbp*^f/f^ (Zhang et al., 2004), *Ep300*^f/f^ (Kasper et al., 2006), *Crebbp*^+/−^ (Tanaka et al., 1997), and CaMKIIα-creERT2 (Erdmann et al., 2007) mice have been previously described. We generated tamoxifen (TMX)-regulated, forebrain-restricted KO (referred to as ifKO) by crossing *Camk2*α-creERT2 and the corresponding floxed strains (Fig. 1*a*). CBP-ifKO mice and p300-ifKO mice express truncated proteins (CBP^stop523^ and p300^stop587^, respectively) that lack KAT activity and other critical domains involved in protein–protein interactions (Lipinski et al., 2020). The genetic background of all mice was C57BL/6J. Analyses were conducted in young adult (3- to 6-month-old) mice, except for those conducted in aged (15- to 20-month-old) mice (seen Fig. 3). Recombination of floxed alleles was induced by five intragastrical administrations of TMX (20 mg/ml dissolved in corn oil; Sigma-Aldrich) on alternate days (Fiorenza et al., 2016). In all our experiments with ifKOs, we used CaMKIIα-creERT2^–^ littermates treated with tamoxifen as controls. To induce status epilepticus (SE) by kainic acid (KA), we used two different protocols, as indicated: a single intraperitoneal injection of 25 mg/kg or progressive administration starting with 5 mg/kg and injecting 2.5 mg/kg with 20 min intervals until the animal reached twice the level 4 in the Racine scale or level 5 once (Umpierre et al., 2016). In the kindling experiment, we injected the mice with 45 mg/kg pentylenetetrazol (PTZ) in alternate days and evaluated their behavior during 15 min by using a modified Racine scale. In the enriched environment (EE) experiments, standard housing consisted of 30 × 15 × 11 cm clear cages occupied by up to five mice, while EE boxes were large white Plexiglas boxes (70 × 150 × 30 cm) occupied by up to 15 mice. We used natural materials, plastic tubing, running wheels, and toys to create an EE whose configuration was modified every 48 h shortly before the start of the dark cycle. Mice were maintained and bred under standard conditions, consistent with Spanish and European regulations and approved by the Institutional Animal Care and Use Committee.

### Behavior

**Figure 2.**
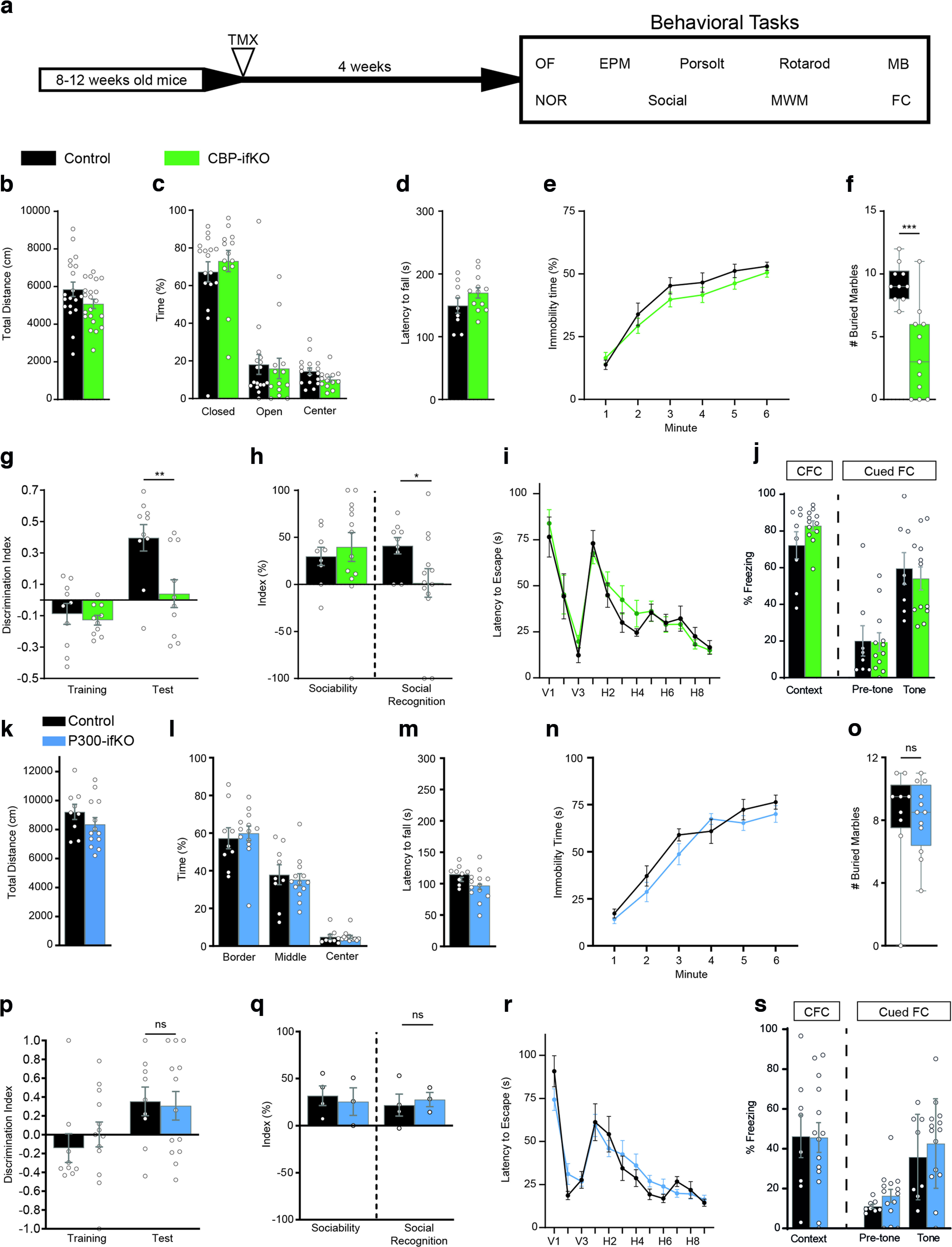
The loss of CBP, but not p300, in forebrain principal neurons causes cognitive deficits. ***a***, Scheme indicating the age at the time of TMX administration to trigger gene ablation, the interval until behavioral testing, and the battery of behavioral tasks (Extended Data Fig. 2-1, additional detail). Experiments were performed in young adult (3- to 6-month-old) mice. ***b***, Behavior in an open field evaluated as total distance traveled. ***c***, No difference in time spent in the center, closed and open arms in the elevated plus maze test. ***d***, Latency to fall in the RotaRod test. ***e***, Percentage of immobility time in the Porsolt forced swimming task. ***f***, Number of balls buried in the marble-burying task. ***g***, Discrimination index during training and testing in the novel object recognition memory task. ***h***, The sociability index reflects the preference of the animal for interacting with another mouse rather than an object, whereas the social recognition index reflects the preference of the animal for interacting with an unfamiliar mouse in the social recognition memory task. ***i***, Graph presents the daily average latency to find the platform in the MWM task. V, Visible platform; H, hidden platform. ***j***, Performance in the contextual and cued fear-conditioning tasks measured as freezing 24 h after training. ***k***, ***l***, Normal behavior of p300-ifKOs in an open field, no differences in overall activity (***a***) or anxiety measured as time near the wall (***b***). ***m***, Normal behavior of p300-ifKOs in the RotaRod. ***n***, Normal behavior of p300-ifKOs in the Porsolt forced swimming task. ***o***, Normal behavior of p300-ifKOs in marble-burying task. ***p***, Normal performance of p300-ifKOs in the novel object recognition task. ***q***, Normal performance of p300-ifKOs in the sociability and social recognition task. ***r***, p300-ifKOs show no differences in learning and memory in the MWM. The panel shows only the latency curve, but similarly no difference was observed in different parameters during the visible and hidden platform tasks and the probe trials. ***s***, Normal performance of p300-ifKOs in the fear conditioning task. ns: non significant; *p-value < 0.05; **0.001 < *p*-value < 0.01; ***p-value < 0.001.

For behavioral testing, we used adult mutant and control littermates of both sexes, except in the case of the EE experiments that were conducted exclusively with females to prevent the frequent fighting between males housed in an enriched environment. The open field (OF), elevated plus maze (EPM), and fear-conditioning tasks were performed as previously described (Viosca et al., 2009, 2010). The Porsolt and marble-burying (MB) tasks were also performed as described previously (Scandaglia et al., 2017). To study motor coordination and learning, mice were trained on a RotaRod (Ugo Basile) at a constant speed (4 rpm) four times per day during 2 d. In the novel object recognition (NOR) memory task, the procedure was similar to that described by Puighermanal et al. (2009): mice were habituated for 10 min to a white acrylic box (48 × 48 × 30 cm) 1 d before the training session. The next day, mice were exposed to a 10 min training session, and 24 h later to a 10 min test session. A three-chambered social test was performed as previously described (Ito et al., 2014). Mice were placed in the center chamber with access to both side chambers containing one empty cylinder and were allowed to freely explore the arena for 5 min (Hab). Then, a mouse with the same sex and age than the tested mouse (cage mate) was placed in one of the two empty cylinders (we systematically alternated left and right cylinders for the locations of the empty cylinder and the cage mate), and we allowed another 10 min of exploration. The interaction with the empty or the occupied cylinder was scored (sociability test). Subsequently, the tested mouse was returned to its home cage and an unfamiliar mouse was introduced into the empty cylinder to assess social recognition. Ten minutes later, the tested mouse was reintroduced in the same context, and we scored the time that the tested mice spent interacting with the familiar and novel mouse during 10 min (social recognition test). Spatial learning in the Morris water maze (MWM) was assessed in a circular tank (diameter, 170 cm) filled with opaque white water as previously described (Viosca et al., 2009). A platform of 10 cm diameter was submerged below the water surface in the center of target quadrant. Mice were monitored with the video-tracking software SMART (Panlab S.L.) during visual sessions (V; 3 d: V1 to V3), when the platform was visible, and during the hidden sessions (H; 9 d: H1 to H9). Each test had a maximum duration of 120 s; if the mouse did not reach the platform it was guided to it. Memory retention probe trials (PTs) of 60 s were performed at the beginning of day H5 (PT1) and 24 h after the last day of the MWM (H10, PT2). The MWM results (see Figs. 2, 8) correspond to experiments in which cohorts of ifKO mice and control littermates housed in a standard cage (SC) or an EE box were evaluated in parallel; the graphs seen in Figure 2 facilitate the comparison between genotypes, whereas those seen in Figure 8 highlight the housing effect on the different genotypes. Regarding statistical analyses, a nonparametric Mann–Whitney *U* test/Wilcoxon rank-sum test was used for pairwise comparison. Training curves were analyzed using repeated-measures ANOVAs including session as the within-subject factor and genotype as the between-subjects factor. Mean ± SEM values are presented in the figures. Additional statistical information for the behavioral testing is provided in Extended Data Figures 2-1, 3-1, and 8-1.

### Magnetic resonance imaging and histologic methods

Perfused mouse heads in agarose were examined in a horizontal 7 T scanner with a 30-cm-diameter bore (model Biospec 70/30 v, Bruker Medical), as previously described (Scandaglia et al., 2017). Nissl staining was conducted as previously described (Lopez de Armentia et al., 2007). In both techniques, the hippocampal area per slice was measured using ImageJ.

### Western blot and immunohistochemistry

Western blot (WB) and immunohistochemistry (IHC) analyses were conducted as previously described (Sanchis-Segura et al., 2009). The following primary antibodies were used in this study: anti-CBP [catalog #sc-583, Santa Cruz Biotechnology (chromatin immunoprecipitation [ChIP], 10 μg)]; anti-CBP [catalog #sc-369, Santa Cruz Biotechnology (WB, 1:500)]; anti-CBP [catalog #sc-7300, Santa Cruz Biotechnology (IHC, 1:100; WB, 1:500)]; anti-p300 [catalog sc-585, Santa Cruz Biotechnology (IHC, 1:100; ChIP, 10 μg)]; acetyl-histone antibodies specific for the pan-acetylated forms of H2A (K5, K9), H2B (K5, K12, K15, K20), H3 (K9, K14), and H4 (K5, K8, K12, K16) produced in our laboratory (WB, 1:500; Sanchis-Segura et al., 2009); commercially available antibodies against H3K9,14ac (06–599), H2B (07–371), H3 (05-499), H2BK15ac (07–343), and H2BK20ac (07–347), from Millipore; H2BK5me2 (catalog #ab17351, Abcam); H2BS14p (07–191), from Millipore; H3K27ac [catalog #ab4729, Abcam (IHC, 1:1000; WB, 1:1000; ChIP, 5 μg)]; anti-NeuN [catalog #MAB377, Millipore (IHC, 1:500)]; anti-GFAP Sigma G9269 (IHC: 1:200; ICC: 1:100); anti-Cre recombinase (Kellendonk et al., 1999; IHC: 1:500); anti-β-actin (F5441; WB, 1:1000). Biotinylated anti-mouse (1:500; catalog #B0529, Sigma-Aldrich) and anti-rabbit (1:3000; B8895, Sigma-Aldrich) antibodies were used in the DAB staining (catalog #11718096001, Sigma-Aldrich). Fluorophore-coupled secondary antibodies were acquired from Thermo Fisher Scientific and used in a dilution 1:400. Cell counting and length measurements were performed using ImageJ software.

### RNA-seq and quantitative real-time PCR assays

Quantitative real-time PCRs (qRT-PCRs) were performed as detailed in the study by Valor et al. (2011). All primer sequences are available on request. Each independent sample was assayed in duplicate and normalized using GAPDH levels. RNA-seq was performed as detailed in the study by Lipinski et al. (2020) with minimal modifications (i.e., single-end libraries; length, 50 bp; HiSeq 2500 Sequencer, Illumina). Information, for example, about the number of replicates and millions of reads per sample is shown in Extended Data Figure 1-1 (see Figs. 1, 7, 9, experiments). Alignment was performed with *STAR* (version 2.6.1) to mm10 (GRCm38) genome and only reads with mapq > 10 were considered for further analysis. Counts were quantified to exons using Rsubread and Mus_musculus.GRCm38.99.gtf from Ensembl. Differential expression analysis was performed with Deseq2 (version 1.30.1). For differentially expressed genes (DEGs) in p300-ifKO mice, each sample corresponded to total RNA from the hippocampus of individual p300-ifKO mice or control littermates (*n* = 3- to ∼4-month-old male mice). For DEGs in CBP-ifKO mice, we conducted the following three independent experiments: (1) each sample corresponded to total RNA from pooled hippocampi of three female mice housed in SCs (*n* = 3- to ∼4-month-old CBP-ifKO mice or control littermates); (2) samples corresponding to total RNA from hippocampus of individual saline-treated male mice in the kindling experiment (see Fig. 7; *n* = 3- to ∼4-month-old CBP-ifKOs or control littermates); and (3) samples correspond to total RNA from hippocampi of individual SC-housed female mice in the EE experiment (see Fig. 9; *n* = 3- to ∼4-month-old CBP-ifKOs or control littermates). For statistical analysis, we used the Wald test in Deseq2 to retrieve DEGs comparing control and CBP-ifKO mice. We identified as bona fide DEGs in CBP-ifKOs those genes retrieved in the three RNA-seq screens. In the RNA-seq screen for kindling-regulated transcripts (see Fig. 7), we also used the Wald in Deseq2 to retrieve DEGs comparing the PTZ45 versus saline conditions in both genotypes. The DEGs in the control group were used to elaborate on the heatmap seen in Figure 7*f*. In the RNA-seq screen for EE-regulated transcripts (see Fig. 9*a*), a likelihood rate test was performed with all the conditions in Deseq2. The design was Genotype + Environment + Genotype:Environment. We selected those genes presenting the main effect for environment and for genotype–environment interaction; outlayer genes were removed by increasing the cooksCutoff to 0.999, and genes with fold > |0.5| were selected.

### ChIP-seq and ChIP assays

ChIP-seq and ChIP-qPCR assays were performed as detailed in the studies by Lopez-Atalaya et al. (2013); Valor et al. (2013b) and Lipinski et al. (2020) using the following antibodies: H2B-ac (K5, K12, K15, K20; Sanchis-Segura et al., 2009), AcH27ac (catalog #ab4729, Abcam), H3K9,14ac (catalog #06-599, Millipore), RNAPII (catalog #sc-9001, Santa Cruz Biotechnology), and TFIIB acetylated at K238 (catalog #ab5210, Abcam). All primer sequences are available on request. ChIP-seq libraries were single-end, 50 bp in length, and sequenced in a HiSeq 2500 apparatus (Illumina). Information about, for example, the number of replicates and the millions of reads per sample is shown in Extended Data Figure 4-1 (Figs. 4, 5, 9, experiments). In the H3K27ac ChIP-seq screen (see Fig. 5), we used datasets from two different experiments, as follows: (1) comparison of samples corresponding to bulk chromatin from the hippocampus of individual (∼4-month-old) CBP-ifKO and control female mice housed in SC or EE (*n* = 2/group; i.e., 4 vs 4 in the genotype-based screen); and (2) samples corresponding to pooled samples from the hippocampi of three CBP-ifKO or control male mice (∼4 months old) housed in SCs (*n* = 2/group). We used the first experiment (with larger sample sizes) to retrieve the differentially acetylated regions (DARs; see Fig. 5*a*,*b*). The H3K27ac-enriched regions retrieved in that screen were used to normalize the second experiment. Both experiments retrieved similar results after normalization. The profiles seen in Figure 5, *a*, *d*, *e*, and *g*, correspond to the second experiment. For the H2Bac ChIP-seq screens, we used bulk chromatin from pooled hippocampi of three CBP-ifKO mice or three control male mice (*n* = 1/group); we also compared samples from *Crebbp*^+/−^ mice and their control littermates (bulk chromatin from pooled hippocampi of three male mice; *n* = 1/group). For the H3K9,14ac ChIP-seq screen, we used bulk chromatin from pooled hippocampi of three CBP-ifKO or three control male mice (*n* = 1/group). For the RNAPII, the ChIP-seq screen used bulk chromatin from the hippocampus of 1 CBP-ifKO or 1 control male mouse (*n* = 1/group). For the TFIIBac ChIP-seq screen, we used bulk chromatin from the hippocampus of 1 CBP-ifKO or 1 control male mouse (*n* = 1/group). For the H3K27ac ChIP-seq screen using sorted neuronal nuclei, the nuclei were isolated from the hippocampus of 1 control female mouse housed in SC or EE box (*n* = 1/group). In addition, we used the following datasets generated in previous studies: ChIP-seq for H3K4me3 (Scandaglia et al., 2017), CBP binding (Lipinski et al., 2020), and assay for transposase-accessible chromatin (ATAC)-seq (Fernandez-Albert et al., 2019) data in wild-type mice were downloaded from Gene Expression Omnibus (GEO; GSE85873, GSE133018, and GSE125068, respectively), and presented in the heatmaps (see Fig. 5*d*,*e*) and the IGV profiles (see Fig. 9*f*). Alignment was done with bowtie2 (version 2.3.4.3). Blacklist regions were removed, and only reads with mapq > 30 and aligned to nuclear chromosomes were used for further analysis. For peakcalling, macs2 was used, and counts were extracted with diffbind (version 3.0.15). Deseq2 was used for the DARs analysis (Fig. 5), using the likelihood rate test and removing the environment effect (EE vs SC) effect. Similar to the description of RNA-seq screen (see Fig. 9), we used Deseq2 and the design Genotype + Environment + Genotype:Environment in selecting regions that present the main effect for environment and for the interaction by selecting those with fold change > |0.25|. To generate normalized coverage tracks, scaling factors for each sample were obtained using Deseq2 sizeFactors. In those cases where samples libraries differences were >5%, we scaled coverages using deeptools and 1/Deseq2 value in the scaleFactor argument. The genomic tracks corresponding to dKAT3-ifKOs ATAC-seq were downloaded from GSE133018 (Lipinski et al., 2020). In all other cases, Reads per Genomic Content was used for normalization. ChipPeakAnno (v 3.20.1) package was used for annotation of ChIP peaks to genomic features. Homer (version 4.10) was used to obtain the number of normalized reads (see Fig. 5*i*, boxplot); these values were plotted using GraphPad, and the Wilcoxon rank-sum test (Wilcox.test one tail with alternative = “greater”) was used for statistical analysis. The selection of 200 random genes expressed in neurons was obtained from the set of genes with more than five transcripts per million in the nuRNA-seq dataset in the study by Fernandez-Albert et al. (2019).

### Other bioinformatic analyses

In both RNA-seq and ChIP-seq experiments, Fastqc (version 0.11.8) was used for quality control of next-generation sequencing (NGS) data, and adapter trimming was performed with trim_galore (version 0.6.4_dev). Reads were aligned to mm10 (GRCm38) as indicated above. Aligned files were processed using samtools (version 1.7) and deeptools (version 3.5.0). IGV (version 2.6.3) was used for visualization and interactive exploration of genomic data. Statistical analyses of RNA-seq and ChIP-seq data were performed in R (version 3.6.1). Gene ontology enrichment analyses were performed with WebGestalt; in the H3K27ac analysis, the weighted set cover option was selected to retrieve the gene ontology (GO) terms.

### Experimental design and statistical analysis

The experimenters were blind to genotypes, and the result of the PCR-based genotyping was provided as a factor for statistical analysis of the behavioral data. All statistical analyses were two tailed except when indicated otherwise. *p* Values or *p*-adjusted values were considered significant at *p* < 0.05 except when indicated otherwise. Mean ± SEM values and percentages are presented in bar graphs. Additional information concerning the experimental design of individual experiments, including the precise statistical tests, and the statistical software used to perform analyses is provided in previous Materials and Methods subsections, an extended data table, and the corresponding figure legends.

### Data availability

The accession number at GEO for the RNA-seq and ChIP-seq datasets reported in this article is GSE200594. In addition, we used the datasets GSE43439, GSE85873, GSE133018, and GSE125068 in specific comparisons.

## Results

### Loss of CBP, but not p300, causes the downregulation of plasticity genes

TMX-treated CBP-ifKO mice showed widespread loss of CBP immunoreactivity in pyramidal and granular neurons in the hippocampus a few weeks after TMX treatment (Fig. 1*a–c*). Other brain regions that also express the Camk2a-creERT2 driver and are known to play a role in learning and memory processes, such as the amygdala and cortex, also lost CBP immunoreactivity, whereas areas in which the driver is not active, such as the cerebellum and the basal ganglia, were spared. Similar results were observed in p300-ifKOs when antibodies against p300 were used (Fig. 1*a*; Lipinski et al., 2020, their Supplementary Fig. S1A,B).

10.1523/JNEUROSCI.0970-22.2022.f1-1 Figure 1-1 RNA-seq samples generated in this study. Download Figure 1-1, XLSX file.

10.1523/JNEUROSCI.0970-22.2022.f1-2 Figure 1-2 Two sheets. ***a***, Downregulated genes in the CBP-ifKO RNA-seq. ***b***, Upregulated genes in the CBP-ifKO RNA-seq (see attached Excel file). Download Figure 1-2, XLSX file.

Although neither CBP-ifKOs nor p300-ifKOs displayed overt neurologic abnormalities after gene ablation, previous studies on conditional CBP KOs have shown that specific forms of neuronal plasticity were compromised by the loss of CBP (Chen et al., 2010; Barrett et al., 2011; Valor et al., 2011; Lipinski et al., 2019). We conducted RNA-seq screens in the hippocampus of CBP-ifKOs, p300-ifKOs, and their control littermates 1 month after inducible gene ablation in adult excitatory neurons to explore the specific impact of eliminating either one of these coactivators at the whole-transcriptome level (Fig. 1*a*, bottom scheme, Extended Data Fig. 1-1). The screen for bona fide CBP-regulated genes (based on three independent RNA-seq experiments) retrieved >200 genes deregulated in the hippocampus of CBP-ifKOs (Fig. 1*d*, top; adjusted *p*-value < 0.05). In contrast, the loss of p300 in mature hippocampal neurons did not cause significant changes in gene expression (Fig. 1*d*, bottom; adjusted *p*-value < 0.05) except for the upregulation of the genes *Arsi* and *Esr1* (also detected in CBP-ifKOs), which is a feature of Camk2a-creERT2 transgenics (*Arsi* is adjacent to the Camk2a promoter and is part of the BAC used to create this Cre-driver strain; the upregulation of *Esr1* is limited to the exons that are part of the creERT2 chimeric construct). GO analysis of the 192 downregulated genes in CBP-ifKOs retrieved a strong enrichment for terms related to neuronal growth, learning and memory, and synaptic transmission (Fig. 1*e*). For instance, several activity-regulated genes encoding important plasticity-related factors (Fernandez-Albert et al., 2019), such as the brain-derived neurotrophic factor (BDNF), neuronal pentraxin (Nptx2), and the neuropeptides enkephalin and dynorphin, were consistently downregulated (Fig. 1*f*, Extended Data Fig. 1-2). Many of the affected genes are included in the group of so-called late response genes (LRGs). The transcripts encoding postsynaptic proteins, such as C1ql2, calmodulin kinases, and serotonin and somatostatin receptors, were also reduced, suggesting that synaptic plasticity is altered in CBP-ifKOs. In contrast, the set of upregulated genes (only 19 genes) was not enriched in any biological process or molecular function. We confirmed some of these changes in independent qRT-PCR assays (Fig. 1*g*).

Note that although the transcriptional dysregulation in CBP-ifKOs affected genes of great relevance in plasticity, the changes were relatively weak when compared with those observed in double ifKOs for CBP and p300 (called dKAT3-ifKOs) in which thousands of genes were severely dysregulated and many changes were greater than twofold (Lipinski et al., 2020; Fig. 1*h*). This comparison indicates that the expression of p300 compensated for the loss of CBP in >95% of the genes (Fig. 1*i*). Furthermore, comparison with a differential expression screen in *Crebbp*^+/−^ mice (Lopez-Atalaya et al., 2011) revealed that the set of genes downregulated in CBP-ifKOs also showed a global trend toward downregulation in hemizygous mice (Fig. 1*j*), thereby underscoring the potential relevance of these transcriptional changes in RSTS etiopathology. Overall, these analyses demonstrate that the expression of a subset of plasticity genes, including genes as relevant as *Bdnf*, *Penk*, and *Nptx2*, is highly dependent on CBP levels.

### Loss of CBP, but not p300, causes cognitive impairments

We next conducted a detailed behavioral characterization of CBP and p300-ifKOs to assess how the regulated ablation of these genes in the adult brain affected multiple behavioral traits (Fig. 2*a*, Extended Data Fig. 2-1). CBP-ifKOs did not show any difference in locomotion or anxiety behavior in an OF (Fig. 2*b*). Anxiety in the EPM task (Fig. 2*c*), motor coordination in the RotaRod (Fig. 2*d*), and depressive-like behaviors in the Porsolt forced swim test (Fig. 2*e*) were also unaffected. However, CBP-ifKOs displayed significantly less MB behavior than their control littermates (Fig. 2*f*). Loss of CBP in the excitatory forebrain neurons also caused impairments in cognitive tasks, such as NOR memory (Fig. 2*g*) and social recognition memory (Fig. 2*h*), while spatial navigation in the MWM (Fig. 2*i*) and contextual and cued fear conditioning (FC) memory were unaffected (Fig. 2*j*). In contrast, the p300-ifKOs did not show any significant difference in locomotion, anxiety, depression, marble-burying behavior, or memory tasks (Fig. 2*k–s*).

10.1523/JNEUROSCI.0970-22.2022.f2-1 Figure 2-1 Extended information on statistical analysis of behavioral experiments in Figure 2. Download Figure 2-1, XLSX file.

Overall, these experiments demonstrate that CBP, but not p300, is required in adult excitatory neurons for optimal performance in different cognitive tasks that rely on spontaneous exploratory behavior. These results extend previous observations in noninducible CBP forebrain-specific KOs (Chen et al., 2010; Valor et al., 2011) and provide conclusive evidence for a pivotal contribution of CBP to adult cognitive function independent of its roles during brain development and maturation.

### Progression of neurologic phenotypes with aging

**Figure 3.**
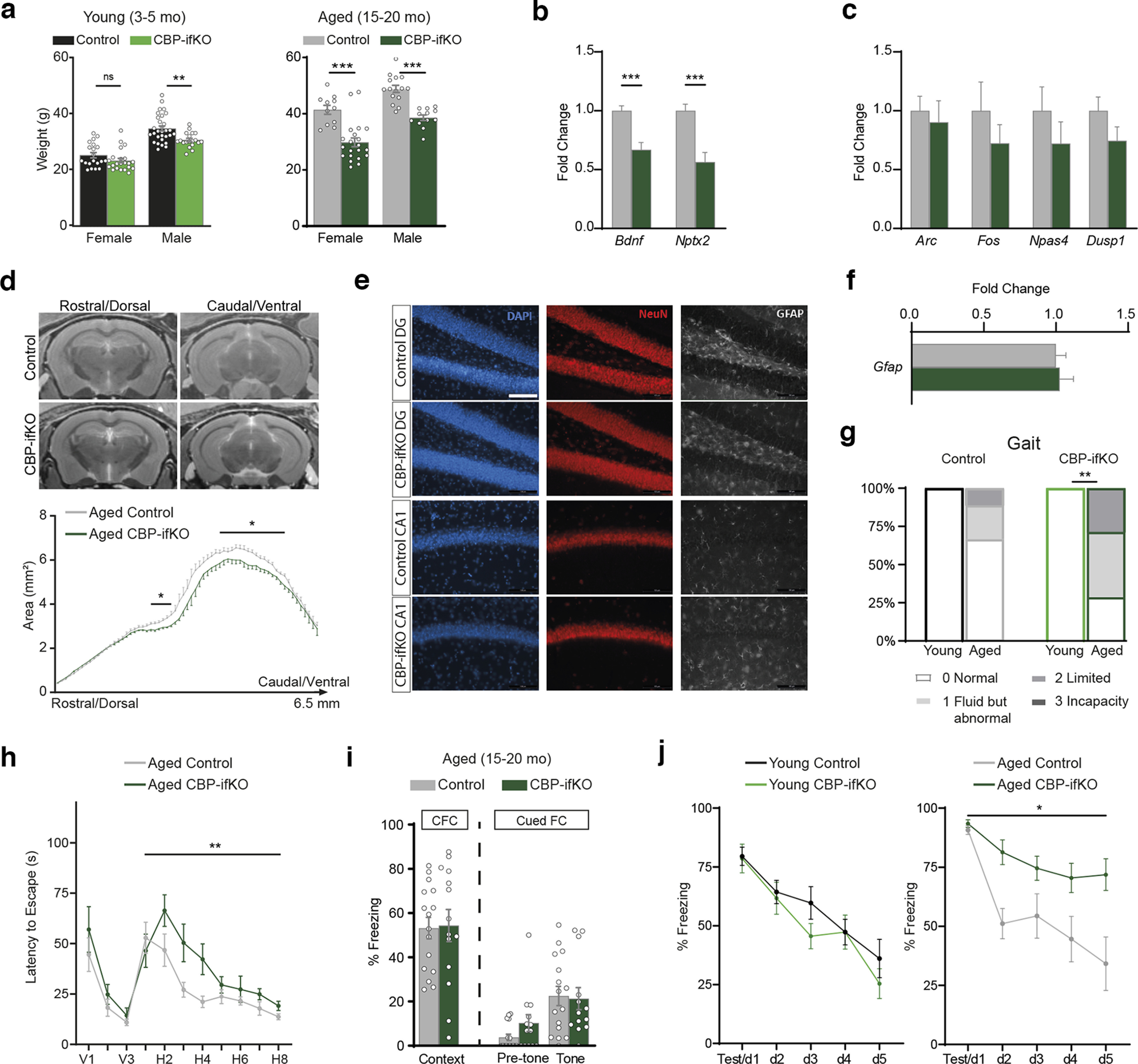
Aging worsens the neurologic deficits in CBP-ifKO mice. ***a***, Average weight in young adult and aged mice of both sexes. ***b***, qRT-PCR assays show the dysregulation of LRGs in aged (15- to 20-month-old) CBP-ifKOs, as previously shown in younger animals (compare Fig. 1*g*, results). ***c***, qRT-PCR assays show that several IEGs were not significantly downregulated at the basal state (Fig. 6*a*, Extended Data Fig. 1-2). ***d***, Representative coronal MR brain images of aged control and CBP-ifKO mice (top) and graph showing the comparison of hippocampal areas between both genotypes (bottom). ***e***, Immunostaining against GFAP and NeuN in the dentate gyrus (DG) and CA1 area of 15- to 20-month-old CBP-ifKOs and control littermates, revealed a normal histology and cellular composition, indicating no apparent gliosis or neurodegeneration in CBP-ifKOs. Scale bars, 100 µm. ***f***, qRT-PCR assay shows comparable levels of *Gfap* transcripts in 15- to 20-month-old CBP-ifKOs and control littermates, consistent with the absence of active gliosis in aged CBP-ifKOs. ***g***, Gait analysis in young adult and aged control and CBP-ifKO mice. ***h***, MWM escape latency graph in aged CBP-ifKOs and control littermates (Extended Data Fig. 3-1, additional detail). ***i***, Memory in the contextual FC (CFC) and cued FC tasks measured as freezing 24 h after training. ***j***, Fear conditioning and extinction in young adult (left) and aged (right) CBP-ifKOs and control littermates. ns: non significant; *p-value < 0.05; **0.001 < *p*-value < 0.01; ***p-value < 0.001.

We extended the neurologic characterization of CBP-ifKOs to older mice. More than 1 year after gene ablation, the reduction in body weight observed in young CBP-ifKO males when compared with control littermates became highly significant and was detected also in females (Fig. 3*a*). Aged CBP-ifKOs, like the younger ones, displayed significant deficits in some LRGs, such as *Bdnf* and *Nptx2*, but not in immediate early genes (IEGs), such as *Arc*, *Fos*, and *Npas4* (Fig. 3*b*,*c*). The chronic downregulation of plasticity genes triggered additional deficits. For instance, the brain of aged CBP-ifKOs showed a moderate atrophy of the ventral hippocampus area (Fig. 3*d*), although we did not detect any apparent neuronal loss (NeuN^+^ cells in CA1 field: Mann–Whitney *U* test = 7, *p* > 0.05 two tailed) or gliosis (Fig. 3*e*,*f*), which suggests a reduction of the hippocampal neuropil that is consistent with our GO analysis (Fig. 1*c*). Moreover, aged CBP-ifKOs displayed behavioral deficits not observed in younger mice, such as gait abnormalities (Fig. 3*g*), spatial learning deficits in the MWM (Fig. 3*h*) and deficient fear memory extinction (Fig. 3*i*,*j*, Extended Data Fig. 3-1). In conclusion, the analyses of elderly mice revealed a progressive deterioration with age, which is consistent with observations in RSTS patients (Stevens et al., 2011), and point to a role for deficient CBP activity in age-related brain pathologies.

10.1523/JNEUROSCI.0970-22.2022.f3-1 Figure 3-1 Extended information on statistical analysis of behavioral experiments in Figure 3. Download Figure 3-1, XLSX file.

### Impaired chromatin acetylation and recruitment of the transcriptional complex at plasticity-related genes

**Figure 4.**
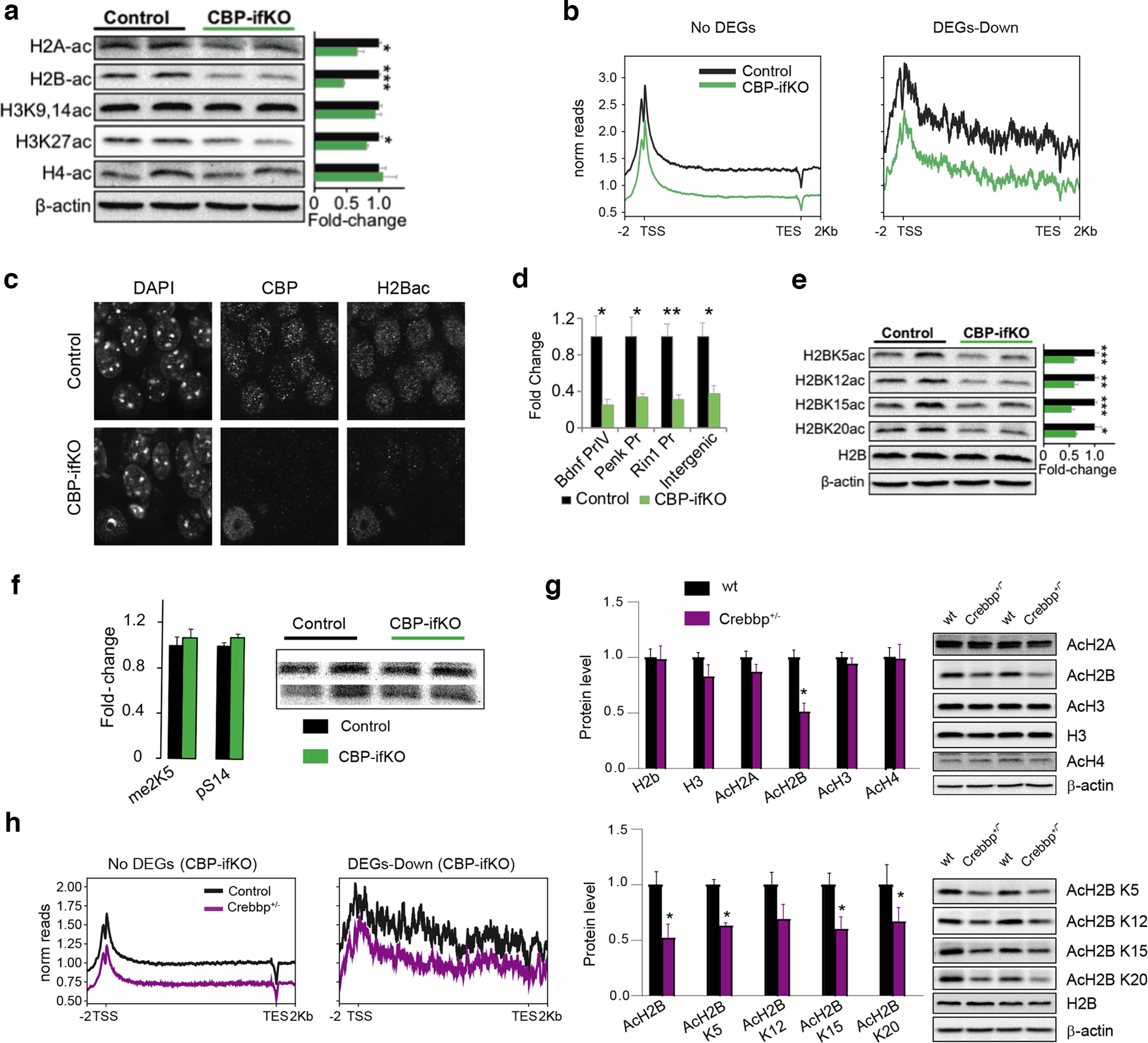
H2B lysine acetylation deficits. ***a***, WB against different core histone acetylations in hippocampal protein extract of CBP-ifKOs and control littermates (*n* = 4). The quantification of the signal is shown on the right. *p-value < 0.05; **0.001 < *p*-value < 0.01; ***p-value < 0.001. A significant decrease in H2A-ac, H2B-ac, and H3K27ac was observed. H3K9,14ac and H4-ac were unaffected by the loss of CBP. ***b***, ChIP-seq was used for the analysis of differential histone acetylation in CBP-ifKOs and control littermates (Extended Data Fig. 4-1, additional detail). Metagene ChIP-seq signal for H2B-ac in the sets of no-DEGs and downregulated genes in CBP-ifKOs. ***c***, IHC for acetylation of H2B in CBP-ifKOs and control littermates. The field shows the nucleus of an interneuron in which creERT2 is not expressed, and therefore there was no loss of CBP immunoreactivity. ***d***, ChIP-qPCR assays on chromatin extracts confirm a global decrease in H2Bac in CBP-ifKOs in all the assessed regions, including intergenic chromatin. ***e***, WB against acetylation of different lysine residues in the N-tail of histone H2B. All the acetylated residues investigated were significantly decreased. The quantification of the signal referred to total H2B is shown in the right bar graph. ***f***, WB against other post-translational modifications of the tail of histone H2B in hippocampal protein extract of CBP-ifKO and control littermates. The quantification of the signal is shown in the bar graph. ***g***, WB against the acetylated form of different core histones (top) and the acetylation of specific lysine residues in the H2B N-tail (bottom) in hippocampal protein extracts of *Crebbp*^+/−^ and wild-type littermates. The quantification of the signal is shown on the right bar graphs. ***h***, ChIP-seq density graph for H2Bac in *Crebbp*^+/−^ and wild-type littermates.

Previous Western blot analyses in *Crebbp*^+/−^ mice (Alarcón et al., 2004; Lopez-Atalaya et al., 2011) and CKIIcre/CBP^f/f^ mice (Valor et al., 2011) have shown that the loss of CBP causes a reduction of neuronal lysine acetylation that differentially affected specific residues in the nucleosome histone tails. We extended these analyses to the brains of CBP-ifKOs and found lysine acetylation deficits that affected histones H2A, H2B, and H3K27 (Fig. 4*a*).

10.1523/JNEUROSCI.0970-22.2022.f4-1 Figure 4-1 ChIP-seq samples generated in this study. Download Figure 4-1, XLSX file.

To link deficient lysine acetylation with transcriptional changes, we extracted hippocampal chromatin from CBP-ifKO and control siblings and performed ChIP-seq against two histone post-translational modifications (hPTMs) that are particularly sensitive to CBP loss: H2Bac and H3K27ac (Alarcón et al., 2004; Valor et al., 2011; Weinert et al., 2018; Lipinski et al., 2020; this study). In parallel, we also examined the biacetylation of histone H3 at K9 and K14, which is in principle not targeted by CBP (Weinert et al., 2018; Extended Data Fig. 4-1).

CBP-ifKOs presented a dramatic and global reduction in H2Bac, an hPTM previously linked with learning and memory (Bousiges et al., 2010), along the entire genome (Fig. 4*b*). This global decrease—manifested in the dramatic reduction of H2Bac levels observed by Western blot (Fig. 4*a*), immunohistochemistry (Fig. 4*c*), and ChIP assays (Fig. 4*d*)—led to a paradoxical increase of H2Bac in promoter regions in ChIP-seq before normalization. This pattern is expected when the changes of a broad-domain histone modification occur at the genome scale (Bonhoure et al., 2014; Orlando et al., 2014; Egan et al., 2016; Guertin et al., 2018). Independent assessment of the four lysine residues in the N-terminal tail of H2B (K5, K12, K15, and K20) by Western blotting confirmed the global hypoacetylation (Fig. 4*e*). Other post-translational modifications of histone H2B, such as phosphorylation and methylation, were, however, not affected by CBP deficiency (Fig. 4*f*). Western blots and ChIP-seq experiments revealed similar H2B acetylation deficits in *Crebbp*^+/−^ mice (Fig. 4*g*,*h*). These results prove that the H2B acetylation deficits first reported in hemizygous mice (Alarcón et al., 2004), which narrowly model the RSTS condition, are not the indirect consequence of developmental effects but are the result of the reduced presence of CBP in the adult neurons. In contrast, our previous analyses in *Ep300*^+/−^ (Viosca et al., 2009, 2010) and p300-ifKO (Lipinski et al., 2020) mice did not reveal H2Bac deficits.

**Figure 5.**
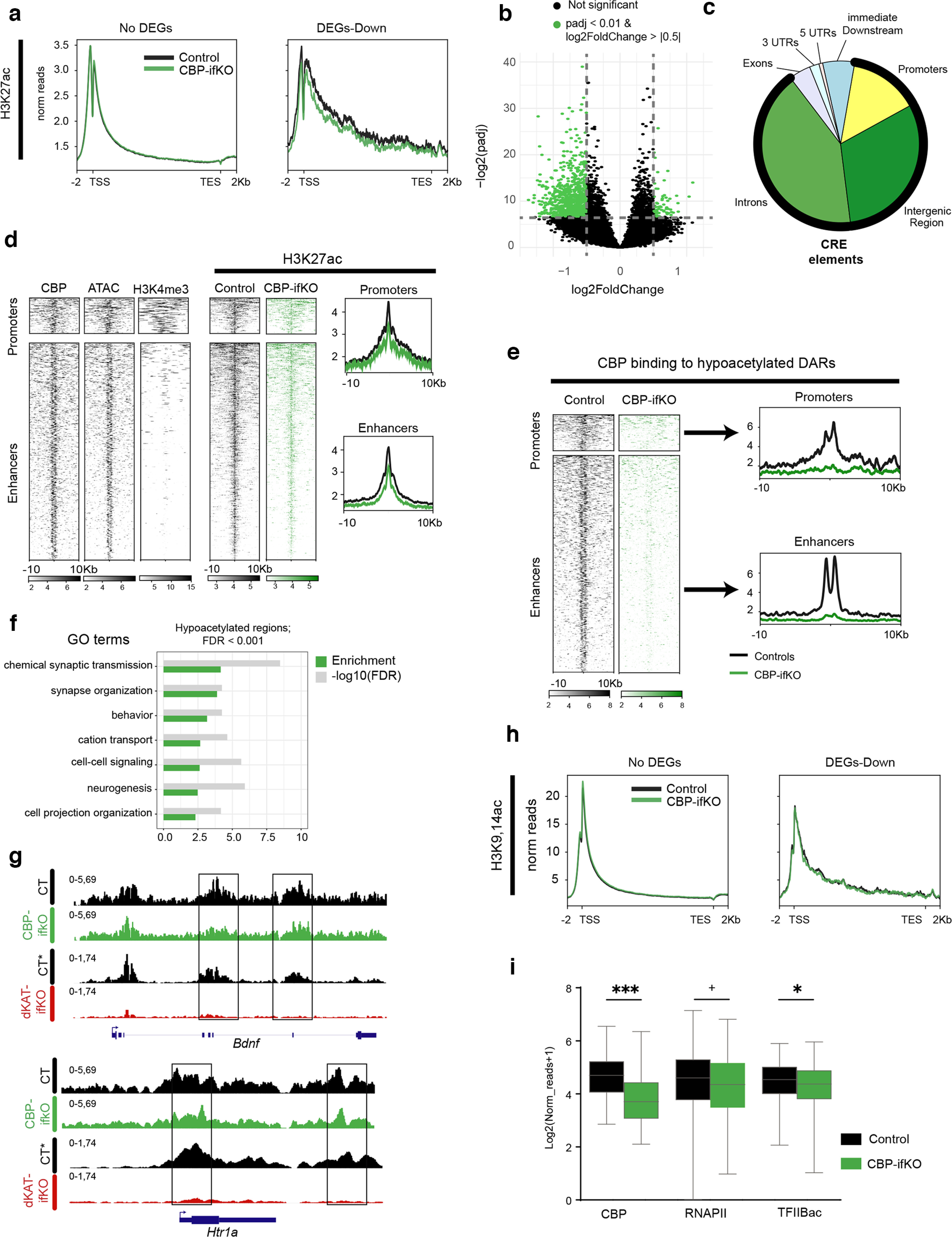
Transcriptional changes are associated with H3K27 lysine acetylation deficits. ***a***, Metagene ChIP-seq signal for H3K27ac in the sets of no-DEGs and downregulated genes in CBP-ifKOs. ***b***, Volcano plot for H3K27-DARs in CBP-ifKOs. We filtered out the regions that match with the BAC construct used to generate the Camk2a-creERT2 transgenics (in those regions, the increase in reads corresponds to changes in gene dose rather than hyperacetylation). ***c***, Positional annotation of hypoacetylated DARs in CBP-ifKOs. Regions with reduced acetylation are primarily located at regulatory regions (CREs; thick black line; number of samples: control = 4, CBP-ifKO = 4). ***d***, Heatmaps show the density of reads in CBP binding, chromatin accessibility (ATAC-seq) and H3K4me3 profiles of control mice and the loss of H3K27ac in DARs of CBP-ifKOs. ATAC-seq and CBP-ChIP signals denote a regulatory role, while H3K4me3 primarily labels promoter regions. ***e***, Heatmap and density profiles of the binding of CBP in CBP-ifKOs and control littermates at decreased DARs. The very weak CBP signal in total hippocampal chromatin of CBP-ifKOs suggests that these regions are neuronal specific. ***f***, Top GO terms from the analysis of hypoacetylated DARs. ***g***, IGV snapshots of H3K27ac ChIP-seq profiles in CBP-ifKOs, dKAT3-ifKOs, and their respective control littermates (CT and CT*, respectively). Two representative genes downregulated in CBP-ifKOs are presented. ***h***, Metagene ChIP-seq signal for H3K9,14ac in the sets of no-DEGs and downregulated genes in CBP-ifKOs. ***i***, ChIP-seq signal for CBP, RNAPII, and TFIIBac at the transcription start site of downregulated genes in CBP-ifKO and control littermates. The observed decreases correlate with the reduced transcription in these genes in mutant mice. + *p*-value < 0.1; *p-value < 0.05; **p-value < 0.01; ***p-value < 0.001.

The analysis of H3K27ac, which typically decorates active enhancer regions (Tie et al., 2009), did not show the same global effect observed in H2Bac, but revealed significant losses at specific regions. We observed a decrease in H3K27ac in the set of downregulated genes retrieved in the RNA-seq screen (Fig. 5*a*). Moreover, when we looked for DARs independently of the expression data (Fig. 5*b*), we found that most of these DARs were located into *cis* regulatory elements (CREs; Fig. 5*c*), being the putative enhancers, the region type that displayed the largest decrease in H3K27ac (Fig. 5*d*). ChIP-seq for CBP in hippocampal chromatin of CBP-ifKOs revealed a pronounced loss of CBP at the affected CREs, indicating that these enhancers are neuronal specific (Fig. 5*e*). Consistent with this result, GO analysis of the genes associated with these DARs retrieved neuronal-specific terms (Fig. 5*f*). These results are consistent with our observations in dKAT3-ifKOs (Lipinski et al., 2020), but the changes observed in the p300/CBP double mutants were stronger and much more numerous than in CBP-ifKOs (Fig. 5*g*). These differences indicate that, although H3K27ac is distinctly dependent on CBP, p300 can maintain functional levels of this hPTM at most loci in the absence of CBP.

Contrary to H2Bac and H3K27ac, the profile for H3K9,14ac was very similar in CBP-ifKOs and control littermates. H3K9,14ac levels were unaffected even in downregulated genes (Fig. 5*h*). These results are consistent with experiments in *Drosophila* and analysis of the CBP/p300-dependent acetylome (Weinert et al., 2018), indicating that these lysine residues are not preferred substrates of CBP.

To shed additional light on defective transcription in CBP-ifKOs, we analyzed the binding of CBP and the RNA polymerase II complex (RNAPII) at downregulated genes using ChIP-seq. RNAPII binding was reduced at the promoter of downregulated genes, concomitantly with CBP binding, H3K27ac levels, and acetylated TFIIB (a subunit of the RNAPII complex regulated by lysine acetylation; Choi et al., 2003; Fig. 5*i*). In contrast, in a set of 200 random neuronal genes, RNAPII and acetylated TFIIB levels were not reduced. These results indicate that the loss of CBP affects the recruitment of the transcriptional complex at neuronal genes.

Overall, these genomic screens and analyses underscore the value of H2B acetylation as a reliable marker for CBP deficiency (including pathologic conditions such as RSTS) and the relevance of H3K27 hypoacetylation in transcriptional dysregulation. They also helped to identify genes particularly sensitive to reduced CBP KAT activity that could be used as biomarkers in pathologies in which this protein has been involved, such as Huntington's disease, Alzheimer's disease and aging-related cognitive decline (Valor et al., 2013a; Achour et al., 2015; Chatterjee et al., 2018; Hervás-Corpión et al., 2018).

### Loss of CBP dampens activity-driven transcription

**Figure 6.**
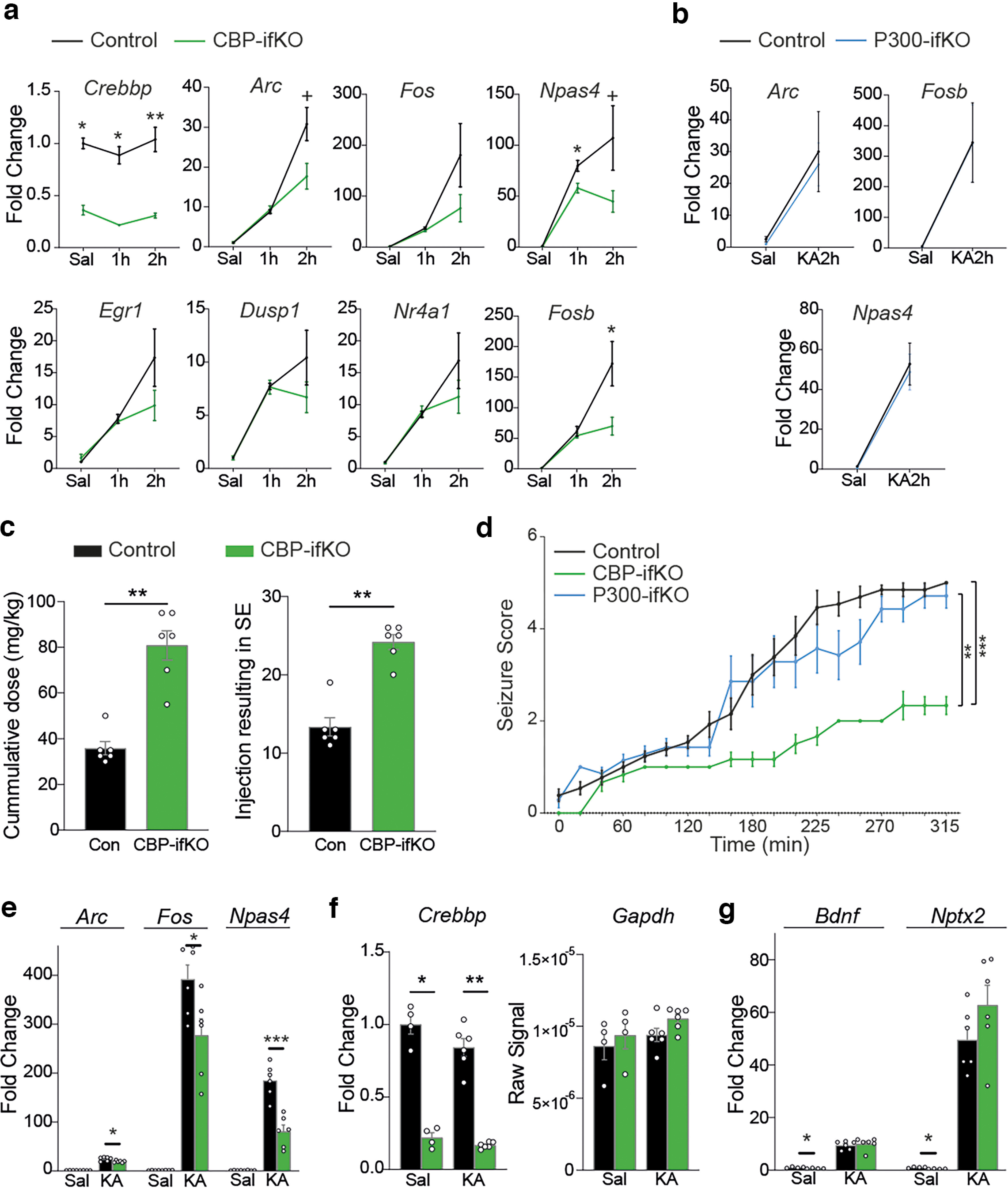
CBP loss impairs activity-driven gene induction. ***a***, qRT-PCR assays show that the initial transcriptional response of CBP-ifKOs to KA is not affected or only slightly reduced 1 h after KA administration, whereas the late response (2 h) is more affected. Asterisks refer to the comparison between genotypes. ***b***, qRT-PCR assays show that IEGs are normally induced by KA in p300-ifKOs. ***c***, CBP-ifKOs require a much larger cumulative dose (left) and number of injections (right) of KA to display seizures. ***d***, Reduced seizure scoring in CBP-ifKOs after progressive KA administration. ***e***, The transcriptional response of IEGs after seizure induced by progressive KA administration is reduced in CBP-ifKOs. ***f***, Similar expression of *Crebbp* and *Gapdh* in CBP-ifKO treated with saline or KA. ***g***, qRT-PCR assays show the reduced expression of *Bdnf* and *Nptx2* in CBP-ifKOs at the basal state, but not after clonic seizure. ^+^*p*-value < 0.1; *p-value < 0.05; **0.001 < *p*-value < 0.01; ***p-value < 0.001.

Our analyses indicate that plasticity-related genes are more dependent on CBP than other genes. Many of these genes are highly expressed in neurons and show a characteristic temporal expression pattern, in which periods of low activity alternate with strong transcriptional outburst. This pattern may require more KAT activity than other genes to maintain the competence of the locus. We hypothesized that the impact of the absence of CBP might be more notorious on activity-dependent bursting of transcription and concurrent histone replacement, which would cause a depletion of acetylation. To test this hypothesis, we evaluated the transcriptional response triggered during SE resulting from the synchronous activation of hippocampal neurons. This includes a rapid induction of IEGs (e.g., *Arc*, *Fos*, *Fosb*, *Npas4*) that is followed by a delayed second wave that includes LRGs, such as *Bdnf*, *Nptx2*, and *Frmpd3*. A single dose of the glutamate agonist KA (25 mg/kg) caused SE and triggered a similar induction of IEGs 1 h after KA administration in CBP-ifKO and control littermates. However, all the tested IEGs displayed lower levels in CBP-ifKOs at later time points, indicating that the transcriptional burst is shorter or weaker in the absence of CBP (Fig. 6*a*). This deficit, like others described in previous figures, was not observed in p300-ifKOs (Fig. 6*b*).

Next, we examined a protocol for seizure induction based on the repeated administration of small doses of KA (2.5 mg/kg every 20 min). In this protocol, control mice needed ∼100 min to develop clonic seizures, whereas CBP-ifKOs showed a delayed (Fig. 6*c*) and attenuated response manifested both in a lower seizure score (Fig. 6*d*) and a weaker induction of IEGs (Fig. 6*e*). qRT-PCR assays confirmed the downregulation of second-wave genes in CBP-ifKOs at the basal state, and revealed no difference in their induction after KA treatment (Fig. 6*f*,*g*). These results indicate that although CBP is not essential for IEG induction, it controls the magnitude of the transcriptional burst. The damped induction of IEGs together with the downregulation of other plasticity genes at the basal state could explain the behavioral deficits observed in CBP-ifKOs.

### CBP loss prevents kindling

**Figure 7.**
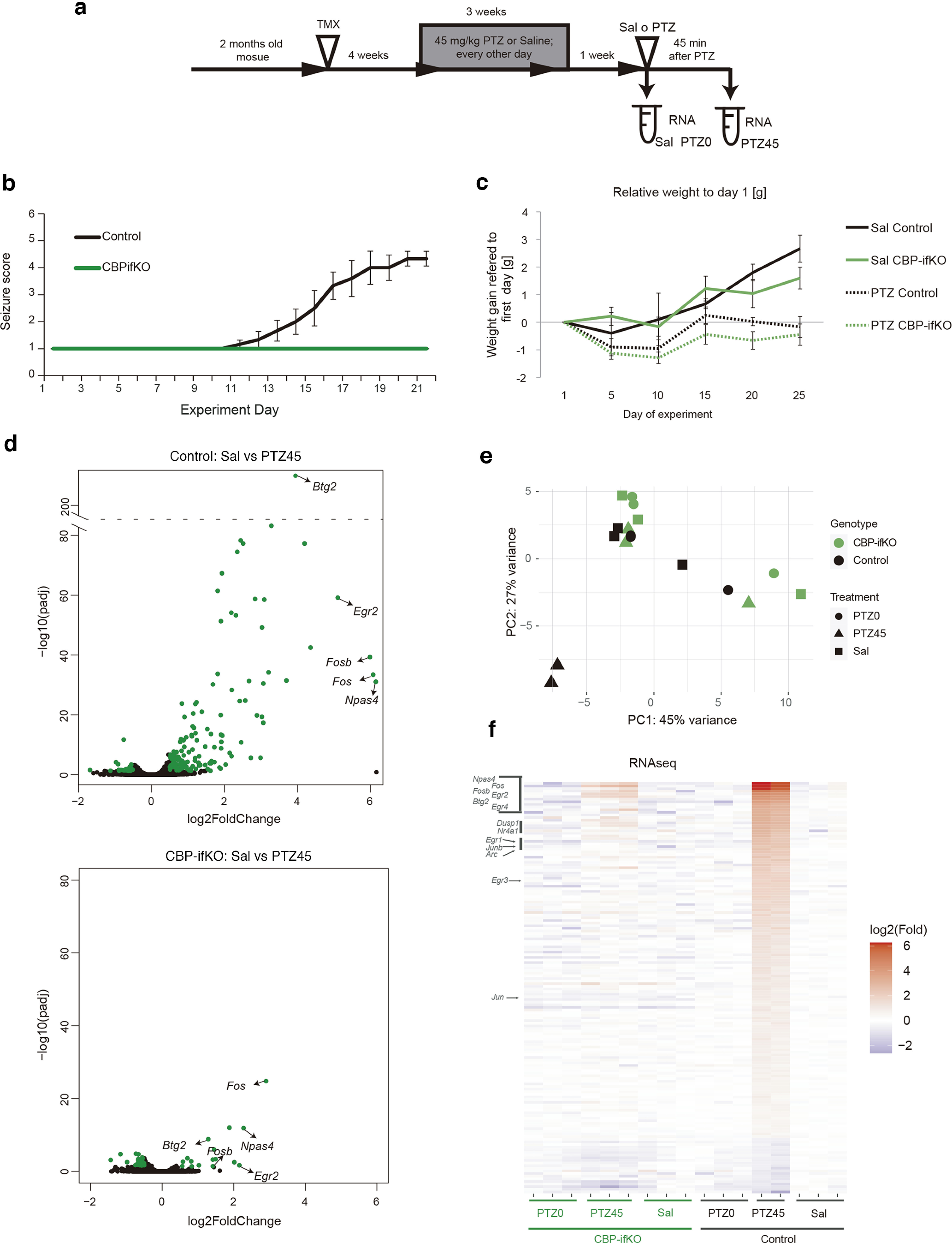
CBP is necessary for kindling. ***a***, Scheme of the kindling experiment. The time when the different RNA-seq samples were taken is indicated. ***b***, Seizure score during the kindling experiment. More details can be found in the Materials and Methods section. ***c***, CBP-ifKOs and control littermates did not gain weight during the kindling protocol. ***d***, Volcano plots presenting the results of the RNA-seq screen performed in control and CBP-ifKO mice. “Sal” corresponds to non-kindled animals treated with vehicle (saline solution) on the day of RNA extraction. “PTZ45” corresponds to kindled animals treated with the PTZ on the day of the experiment. ***e***, PCA for RNA-seq samples of kindling. The control mice treated with PTZ (Ct-PTZ45) induced a gene program that includes dozens of IEGs (Extended Data Fig. 7-1, additional detail). ***f***, Heatmap with the genes showing PTZ induction in the control group (PTZ main effect, 2 × 3 design). In addition to the Sal and PTZ45 samples, “PTZ0” samples correspond to kindled animals treated with saline on the day of the experiment. Transcript levels are shown for all the conditions.

To further test our hypothesis, we challenged CBP-ifKOs in a paradigm that leads to long-lasting changes in response to stimulus: PTZ-induced kindling. In this protocol (commonly used to model epilepsy), an initially subconvulsive dose of PTZ induces progressively more robust seizures after repeated administration (Fig. 7*a*). This sensitization response is likely to rely on transcriptional changes (Perlin et al., 1993; Liang and Seyfried, 2001). Control mice reacted to the treatment as expected, with an initial latent nonconvulsive period, followed by an incremental appearance of seizures. After 10 d of kindling, a subconvulsive dose of PTZ was sufficient to induce seizures in all individuals. To our surprise, none of the CBP-ifKOs entered the progressive seizure stage within >20 d of the experiment. (Fig. 7*b*). Other effects of the treatment, such as the halted gain of weight, were observed in both genotypes, demonstrating that the drug was effectively delivered to KO mice (Fig. 7*c*). We also investigated the transcriptional response associated with kindling in each group. One week after kindling, mice were treated with the same dose of PTZ once again, and 45 min later their hippocampi were extracted for RNA. The RNA-seq analysis revealed the induction of 219 genes in PTZ-treated control mice, whereas CBP-ifKOs showed a much more limited response involving only 62 genes (*p*-adjusted < 0.05; Fig. 7*d–f*, Extended Data Fig. 7-1). IEGs, such as *Fos* and *Npas4*, were induced in both genotypes, but their induction was weaker in CBP-ifKOs. Intriguingly, PTZ-downregulated genes were less affected than upregulated genes, indicating that the anomalous response was largely restricted to gene activation. These results show that CBP is specifically involved in facilitating the transcriptional response to kindling.

10.1523/JNEUROSCI.0970-22.2022.f7-1 Figure 7-1 2 sheets: ***a***, PTZ regulated genes in control mice (comparison PTZ45 vs saline). ***b***, PTZ regulated genes in CBP-ifKO mice (comparison PTZ45 vs saline). Download Figure 7-1, XLSX file.

### CBP loss interferes with the neuroadaptation to environmental enrichment

To further confirm the role of CBP in establishing novel gene programs, we evaluated CBP-ifKOs in another paradigm that triggers enduring changes in hippocampal excitatory neurons: EE. This condition is known to improve the cognitive performance of mice and promote neurogenesis and synaptogenesis (van Praag et al., 2000; Kempermann, 2019). These processes rely on changes in the transcriptome of hippocampal neurons (Fischer, 2016; Grégoire et al., 2018; Zhang et al., 2018). Consistent with our hypothesis, experiments in *Crebbp*^+/−^ mice had revealed an attenuation of the transcriptional program induced by EE (Lopez-Atalaya et al., 2011). However, these deficits could originate from the defective assembly of neuronal circuits because of CBP deficiency during development (del Blanco et al., 2019).

**Figure 8.**
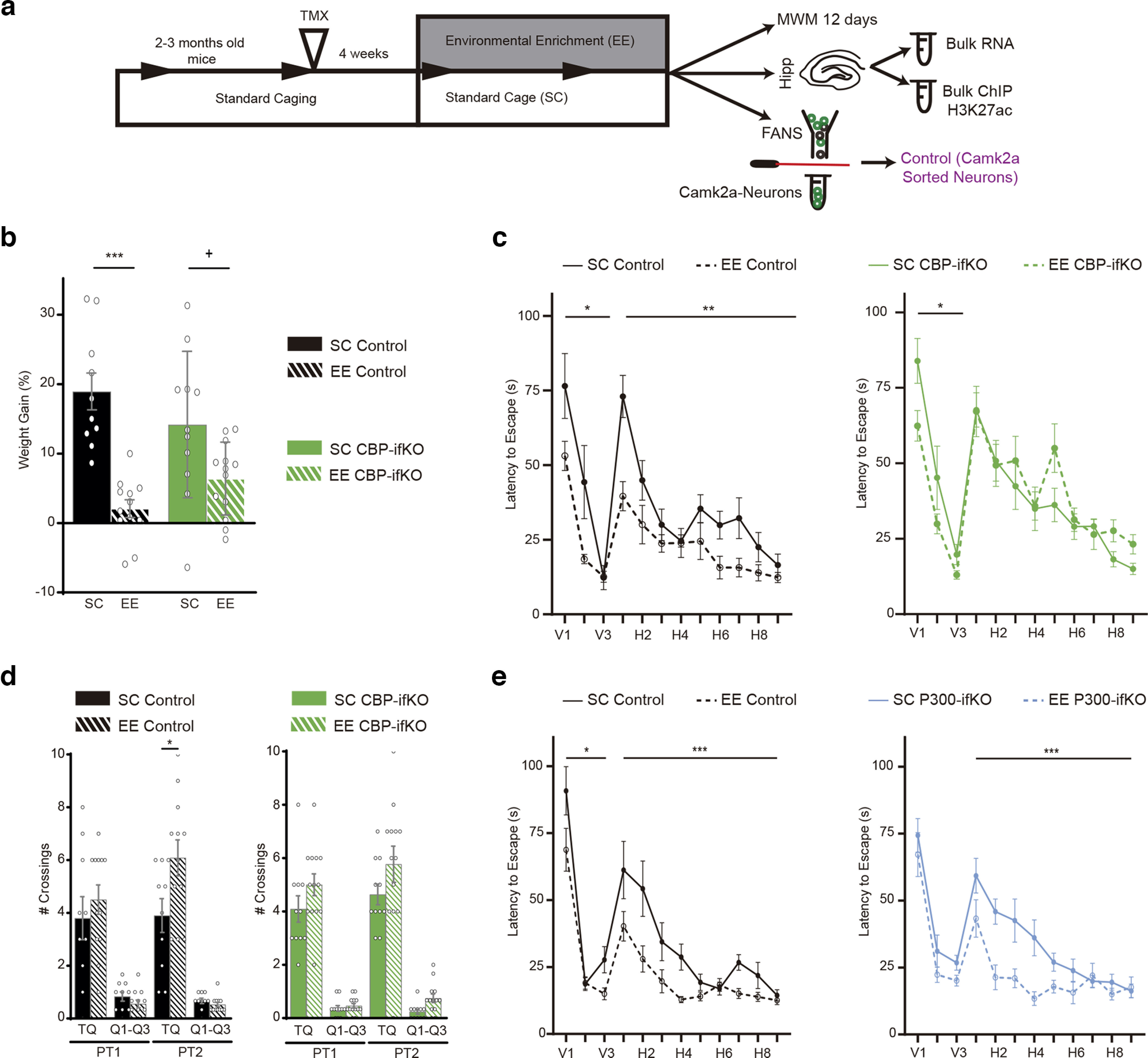
Behavioral impact of EE on CBP-ifKOs and p300-ifKOs. ***a***, Scheme of the EE experiment. CBP-ifKOs and control littermates were housed in SCs or in a large EE box. The time when RNA-seq and ChIP-seq samples were taken is indicated (Extended Data Fig. 8-1, additional detail). ***b***, Control and CBP-ifKO mice exhibit a reduced weight gain when housed in EE cages. ***c***, Latency to find the platform in the MWM task. The graphs compare the performance of control and CBP-ifKOs housed in SCs (solid lines; control, *n* = 10; CBP-ifKO, *n* = 11) or EE (dashed lines; control, *n* = 12; CBP-ifKO, *n* = 13). Note that the curves for the SC groups were previously presented in Figure 2*i* to compare the performance between genotypes. ***d***, Memory retention PTs of 60 s were performed at the beginning of day H5 (PT1) and 24 h after the last day of the MWM (PT2). Graphs show the number of platform crossings during the PTs. TQ, target quadrant; Q1–Q3, quadrants 1 to 3. Comparison between SCs (solid bars) and EE boxes (dashed bars) for controls and CBP-ifKOs. ***e***, MWM results of p300-ifKOs and their control littermates housed in SCs (solid lines; control, *n* = 9; p300-ifKO, *n* = 13) or EE boxes (dashed lines; control, *n* = 10; p300-ifKO, *n* = 7). Graphs represent the time to find the platform. No difference in p300-ifKO performance was observed either in SCs or EE boxes. The curves for the SC groups were previously presented in Figure 2*r*. + *p*-value < 0.1; *p-value < 0.05; **0.001, *p*-value < 0.01; ***p-value < 0.001.

To specifically investigate the interaction between CBP loss and EE in the adult brain, we exposed CBP-ifKOs to EE for 1 month and examined their performance in the MWM, a cognitive task highly sensitive to EE (Fig. 8*a*, Extended Data Fig. 8-1). Mice exposed to EE did not gain weight when compared with animals housed in SCs, likely reflecting the more intense physical activity in EE conditions (Fig. 8*b*). Differences between the two genotypes emerged when we tested the animals in the MWM. Control mice in EE boxes exhibited better learning of the platform location and performed better in the probe trial than those housed in SCs (Fig. 8*c*,*d*). In contrast, CBP-ifKOs exposed to EE did not show significant improvements. To explore the specificity of the CBP function in EE adaptation, we also compared the performance of p300-ifKOs and control littermates housed in SCs or EE boxes and found that in this case both genotypes showed a significant improvement in spatial navigation after EE (Fig. 8*e*). These results demonstrate that CBP plays a unique role as mediator of EE benefits in the adult brain that is not shared with p300.

10.1523/JNEUROSCI.0970-22.2022.f8-1 Figure 8-1 Extended information on statistical analysis of behavioral experiments in Figure 8. Download Figure 8-1, XLSX file.

**Figure 9.**
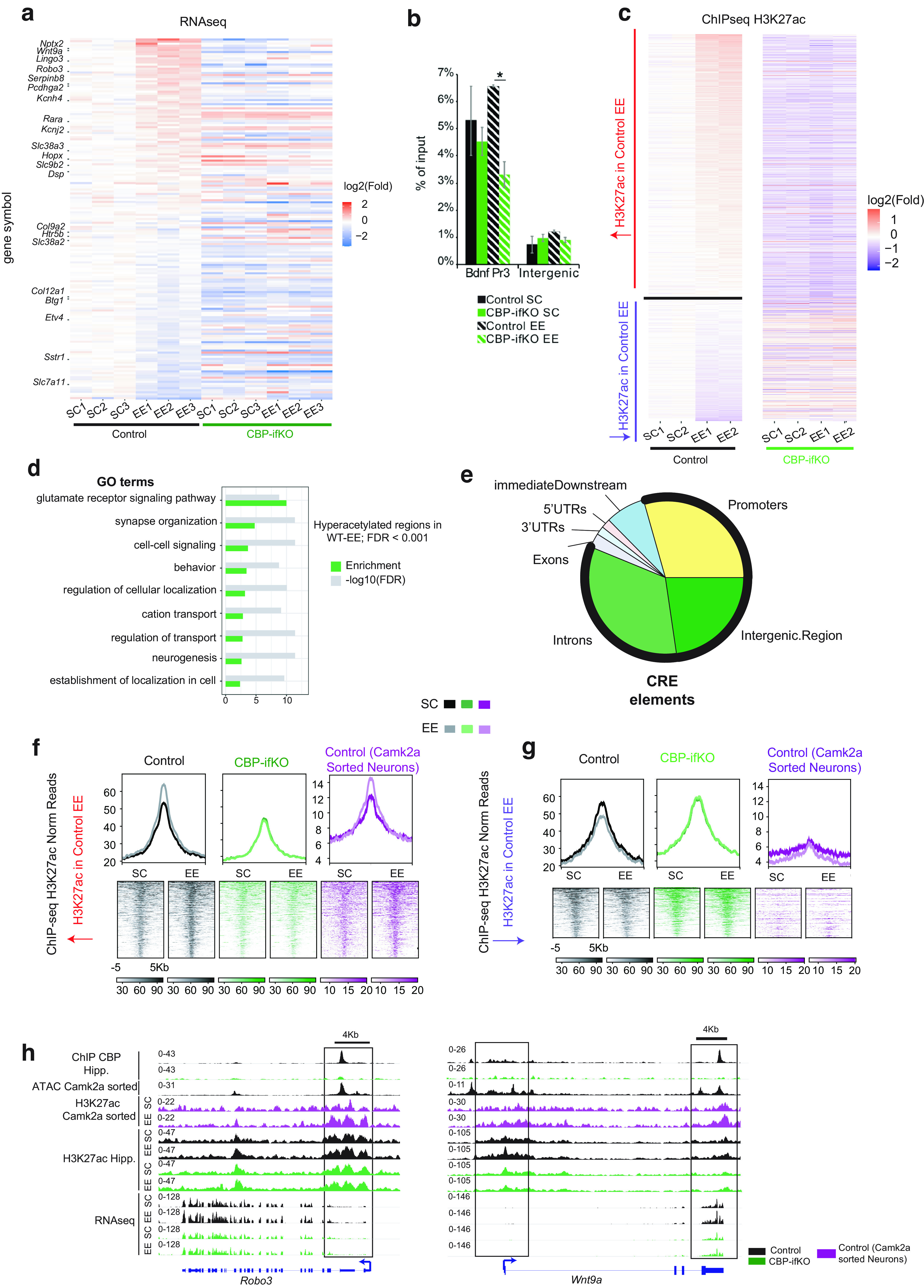
CBP is necessary for neuroadaptation to environmental enrichment. ***a***, Heatmap represents DEGs in the RNA-seq screen showing a significant EE main effect or interaction with genotype (Extended Data Fig. 9-1, additional detail). ***b***, H3K27ac ChIP-qPCRs results at the *Bdnf* locus in the chromatin of SC-housed and EE-housed mice from both genotypes (Extended Data Fig. 9-1, additional detail). ***c***, DARs displaying increased or reduced H3K27ac levels. The changes induced by EE in the control group were not reproduced in CBP-ifKOs. ***d***, GO analysis of DARs that gain H3K27ac on EE-retrieved terms related with synaptic signaling and plasticity. ***e***, Positional annotation of hyperacetylated DARs in EE boxes. Regions with increased acetylation are primarily located at regulatory regions (CREs; thick black line). ***f***, Read density heatmaps show increased H3K27ac in DARs. Regions with increased H3K27ac in EEs are observed in bulk chromatin of control mice, but not in CBP-ifKOs. The increase was also observed in samples corresponding to a H3K27ac ChIP conducted with sorted nuclei from excitatory neurons (in which Camk2a-creERT2 drives the expression of the fluorescent protein Sun1-GFP). ***g***, Read density heatmaps show DARs with reduced H3K27ac in control mice housed in the EE condition. These changes were not detected in CBP-ifKOs. Note that the H3K27ac signal is very low in the ChIP-seq corresponding to sorted neurons from excitatory neurons (in which Camk2a-creERT2 drove the expression of the fluorescent protein Sun1-GFP) when compared with the signal detected in the ChIP-seq using bulk chromatin. The low signal suggests that the H3K27ac signal in these DARs likely corresponds to non-neuronal cells. ***h***, IGV snapshot for *Robo3* and *Wnt9a* showing RNA-seq, H3K27ac and CBP ChIP-seq, and ATAC-seq profiles. *p-value < 0.05.

Next, we extracted total RNA from the hippocampi of CBP-ifKOs and control littermates maintained in standard housing or exposed to EE. RNA-seq profiling identified 173 genes affected by EE (*p*-adjusted < 0.05, log2FoldChange > |0.5|, including both genes with a significant EE effect and a housing × genotype interaction; Fig. 9*a*, Extended Data Fig. 9-1). These transcriptional changes were less prominent in CBP-ifKOs than in their control littermates, particularly for upregulations, which suggests that the resilience of CBP-ifKOs to EE may result from their inability to adjust gene expression. Consistent with this view, ChIP assays at *Bdnf* (a locus known to respond to EE) showed a significant increase in H3K27ac levels in control mice housed in the EE when compared with CBP-ifKO littermates (Fig. 9*b*). To further explore this finding, we performed two independent genome-wide screens to detect changes in H3K27ac in response to EE. In the first experiment using bulk chromatin, we observed that EE caused more increases than reductions in H3K27ac (Fig. 9*c*, Extended Data Fig. 9-1). A second ChIP-seq experiment using sorted nuclei from Camk2a^+^ hippocampal neurons (Fernandez-Albert et al., 2019), allowed us to specifically examine those changes in H3K27ac that occurred in excitatory neurons. Interestingly, the GO analysis of the DARs that gained H3K27ac on EE retrieved terms related with synaptic signaling and organization, and behavior (Fig. 9*d*). Furthermore, the comparison of the two screens indicated that most of the regions displaying gains in H3K27ac on EE were located at regulatory regions in neuronal genes (Fig. 9*e,f*), whereas the regions that displayed losses in H3K27ac mostly came from other cell types (Fig. 9*g*). Notably, EE-induced changes in H3K27ac were largely suppressed in CBP-ifKOs (*p*-adjusted < 0.1, log2FoldChange > |0.25|, including both genes with significant EE effect and housing × genotype interaction; Fig. 9*f*). Among the genes that display an increase in both transcripts and H3K27 acetylation levels in response to EE in control mice but not in CBP-ifKOs, we find *Robo3* [a member of the Roundabout (ROBO) gene family that controls neurite outgrowth, growth cone guidance, and axon fasciculation] and *Wnt9a* (a member of the WNT gene family, which regulates synapse formation and maintenance, axonal remodeling, and dendrite outgrowth; Fig. 9*h*). Although both genes have been involved in neurodevelopmental processes, their roles in the adult brain remain largely unexplored.

10.1523/JNEUROSCI.0970-22.2022.f9-1 Figure 9-1 Three sheets: ***a***, EE-regulated genes in CBP-ifKOs and control littermates (including both genes with significant EE effect and housing × genotype interaction). ***b***, DARs displaying increased H3K27ac after EE (including both genes with significant EE effect and housing × genotype interaction). ***c***, DARs displaying decreased H3K27ac after EE (including genes with both significant EE effect and housing × genotype interaction). Download Figure 9-1, XLSX file.

## Discussion

Studies in the last 2 decades have underscored the role of lysine acetylation in neuroplasticity processes, challenging the idea that chromatin regulators are only relevant during development (Gräff and Tsai, 2013; Lopez-Atalaya and Barco, 2014). Here, we refined the gene ablation strategy of previous loss-of-function studies to specifically evaluate the consequences of CBP and p300 ablation in principal neurons in fully mature neuronal circuits. Using this approach, we showed that the ablation of CBP in forebrain excitatory neurons in adult animals compromised their plasticity capabilities, particularly affecting behavioral tasks that rely on spontaneous exploratory behavior, such as novel object recognition and social recognition memory. In addition to these deficits, old CBP-ifKO mice displayed several age-related phenotypes, such as weight loss, impaired gait, spatial navigation, and extinction. In contrast, the elimination of p300 alone did not cause any apparent phenotype. These results are consistent with the weaker phenotype of *Ep300*^+/−^ mice compared with *Crebbp*^+/−^ and the milder symptoms and lower prevalence of RSTS2 [catalog #613684, OMIM (Online Mendelian Inheritance in Man); linked to *Ep300* mutations] compared with RSTS1 (catalog #180849, OMIM; linked to *CREBBP* mutations; Lopez-Atalaya et al., 2014). Note, however, that these results do not exclude a role for p300 in cognitive and plasticity processes. On the contrary, our experiments underscore the critical role of p300 in the absence of CBP because a single functional *Ep300* allele was sufficient to prevent the dramatic neurologic phenotypes observed after combined elimination of CBP and p300 (Lipinski et al., 2020).

Previous pharmacological and genetic experiments have linked CBP with specific forms of memory, particularly NOR (Chen et al., 2010; McQuown et al., 2011; Valor et al., 2011). Why are some forms of memory more sensitive to CBP loss than others? Korzus et al. (2004) suggested that CBP might be particularly important in tasks that recruit an innate behavioral preference for novelty and does not involve an exogenous reinforcers. This view is in agreement with our findings in NOR, social memory, and MB, supporting the idea that stressful reinforcers, such as a swim stress or an electric shock, activate alternative mechanisms that overcome the lack of CBP.

Biochemical experiments have demonstrated the eviction of histones during transcription elongation and the faster turnover of nucleosomes in heavily transcribed genes (Ferrari and Strubin, 2015). A direct consequence of the eviction of histones during transcription—particularly histones H2A and H2B (Kireeva et al., 2002), which are among the most sensitive to CBP loss—is that the epigenetic status of the gene would be transiently weakened and more susceptible to disruption. The absence of CBP could deplete the gene from acetylation marks interfering with the self-maintenance of the locus over time. As a result, deficits would emerge on rapid, chronic, or repeated activation. Consistent with this prediction, a stronger phenotype was observed in CBP-ifKOs in paradigms such as SE, kindling, or EE adaptation in which the neuronal transcriptome needs to readjust after the experience (Delorenzo and Morris, 1999; Mirza et al., 2017). In these paradigms, CBP-ifKOs failed to adapt their behavioral and transcriptional response. Previous observations in other organisms are also consistent with this view. For instance in *Drosophila*, mutants of *nejire* (CBP ortholog) fail to develop a typical long-lasting alcohol tolerance caused by a single alcohol exposure (Ghezzi et al., 2017). In a different example, dietary restriction increases the expression of CBP or its ortholog gene both in the mouse hypothalamus (Moreno et al., 2016) and in *Caenorhabditis elegans* (Zhang et al., 2009). In mice, CBP deficiency impaired the response to EE in the central (Lopez-Atalaya et al., 2011) and peripheral (Hutson et al., 2019) nervous systems. In *C. elegans*, dietary restriction correlated with an increase of the life span and protection from proteotoxicity, and these effects were suppressed by the inhibition of cbp-1 (Zhang et al., 2009). Therefore, although in normal laboratory conditions KATs other than CBP may be sufficient for preserving acetylation levels at most lysine residues, CBP plays a pivotal role in processes in which the neuronal epigenome needs to be edited both during development and in the adult brain. This view and the postulated weakening of acetylation profiles with aging (Peleg et al., 2010; Harman and Martín, 2020) might explain the worsening on the phenotype and the appearance of additional deficits in elderly CBP-ifKOs.

In conclusion, our study identifies CBP as the main KAT responsible for refreshing the acetylation status of plasticity-related genes after neuronal activation, preserving the competence and inducibility of these loci over time. This makes CBP a key component of the molecular machinery that enables phenotypical variation of behavioral and cognitive traits in response to experience and environmental changes. Our results also shed new light on neurologic and psychiatric conditions in which a defective or reduced CBP activity has been reported and plasticity mechanisms are compromised, such as RSTS, substance use disorders, several neurodegenerative diseases, and aging-related cognitive impairments.
